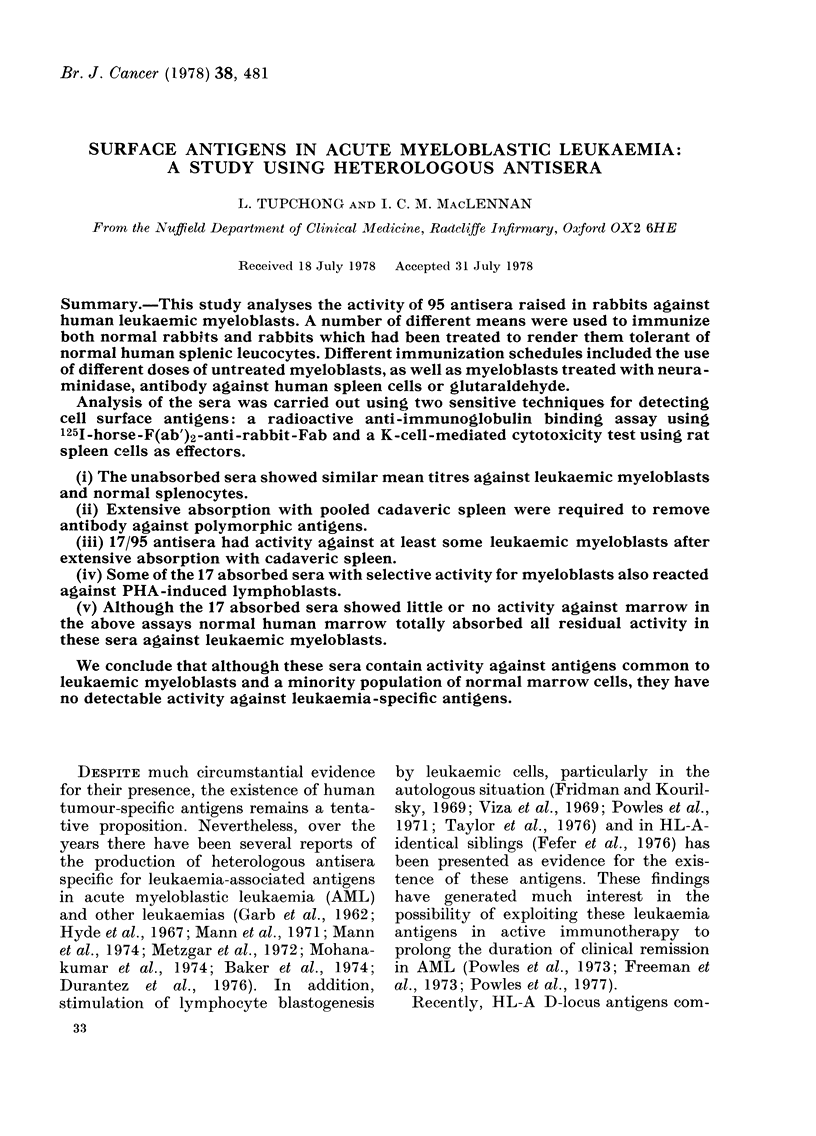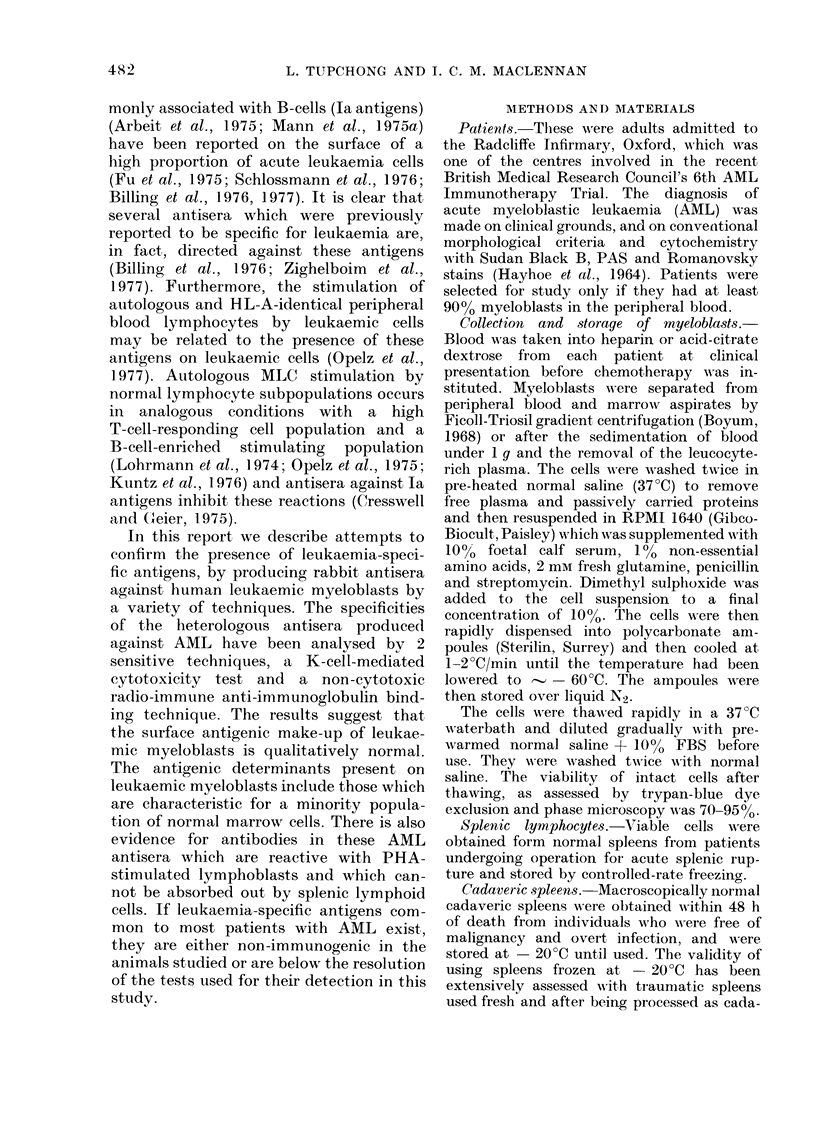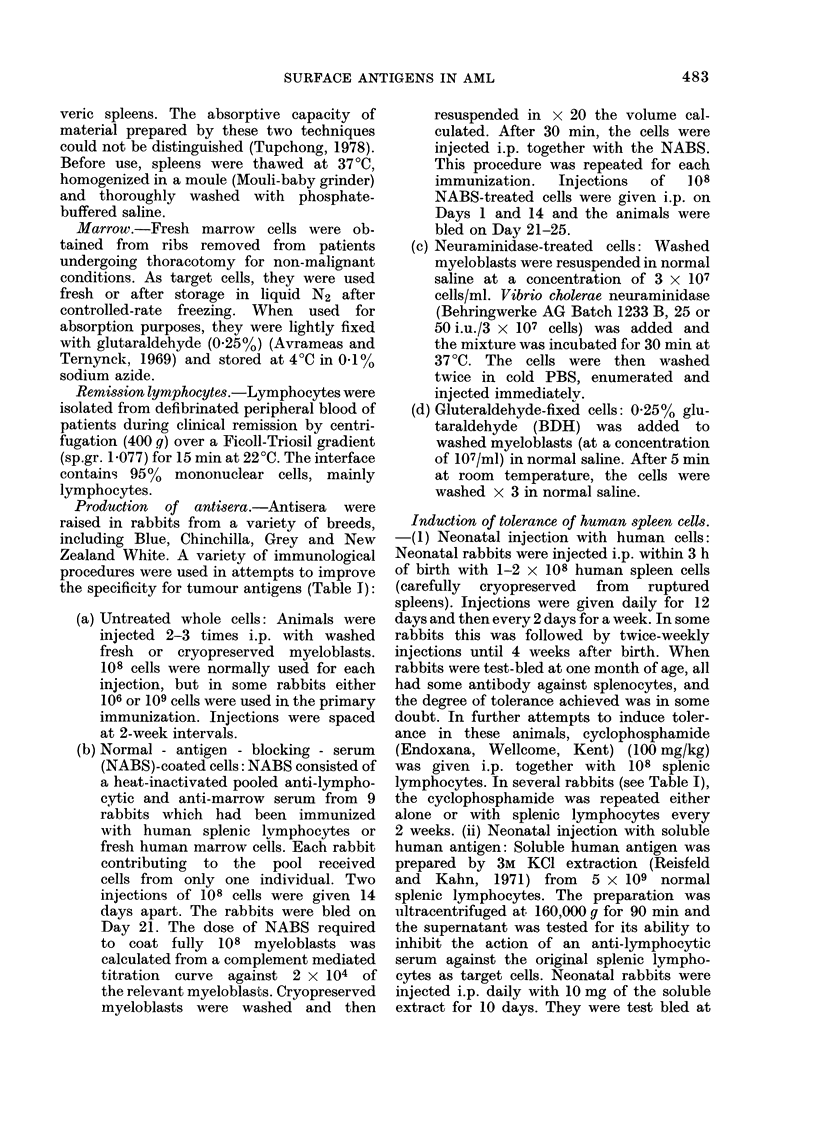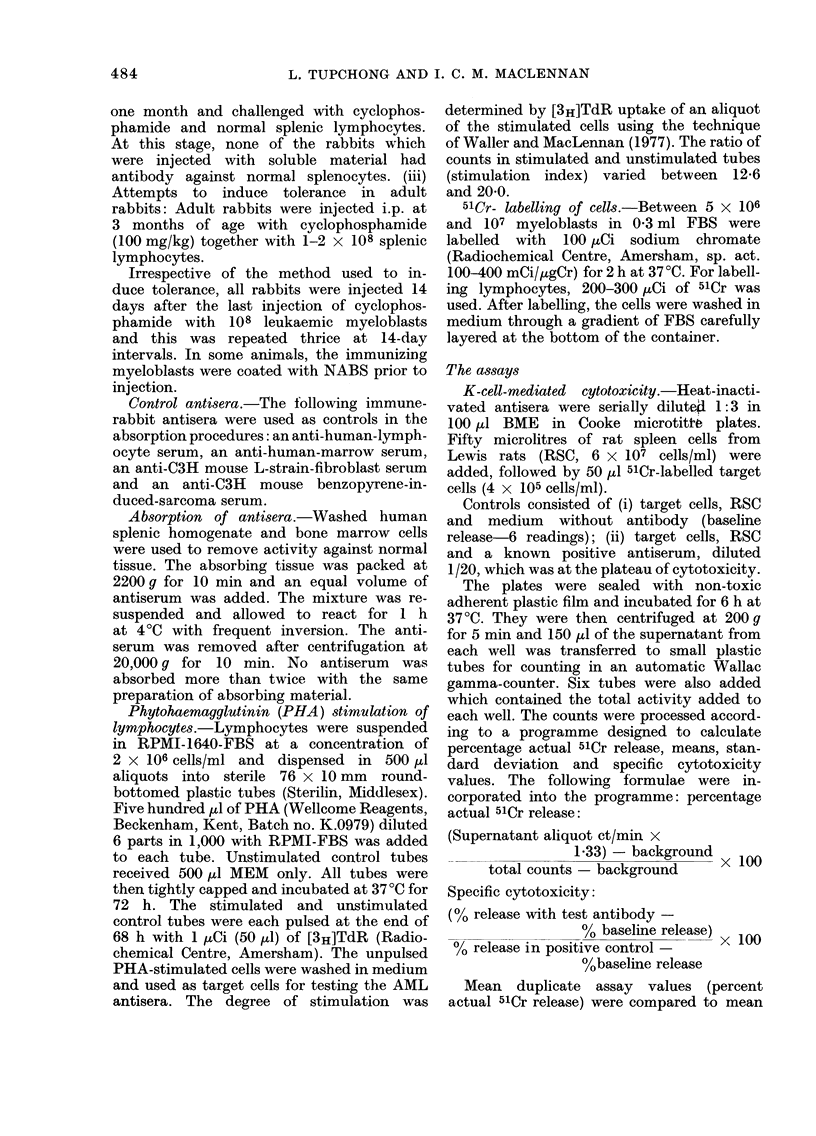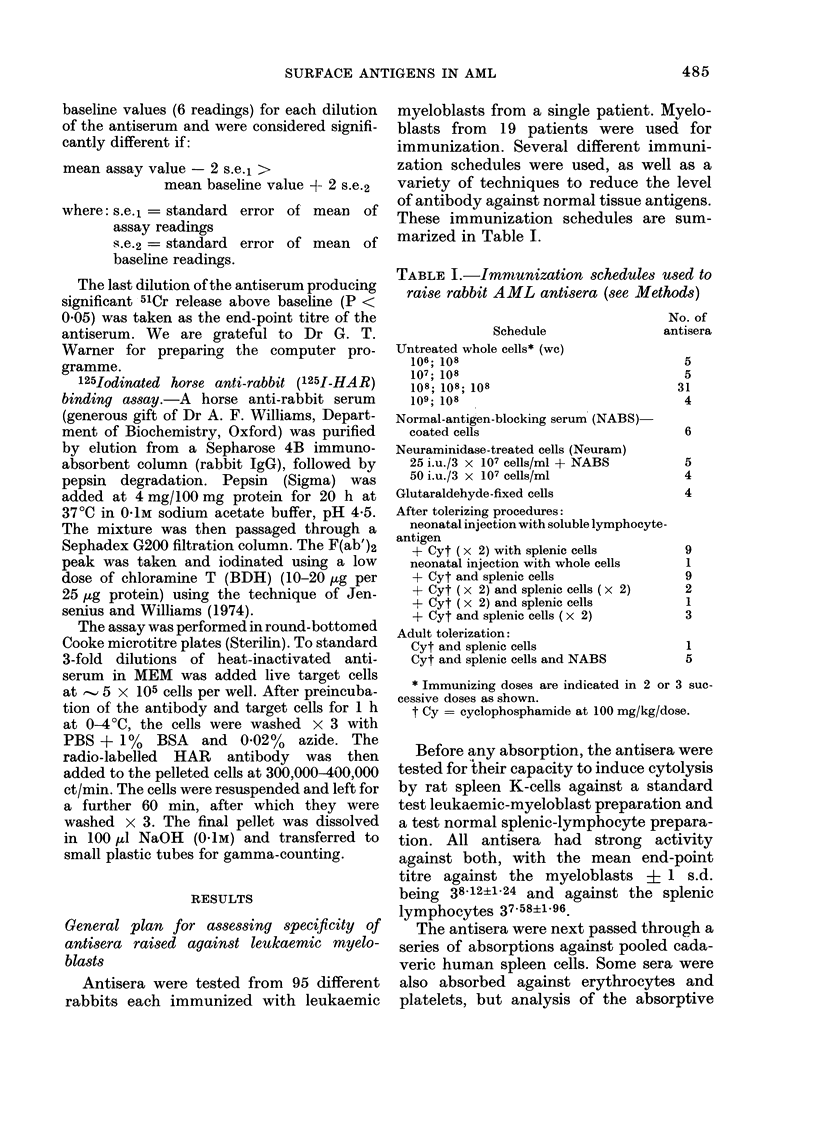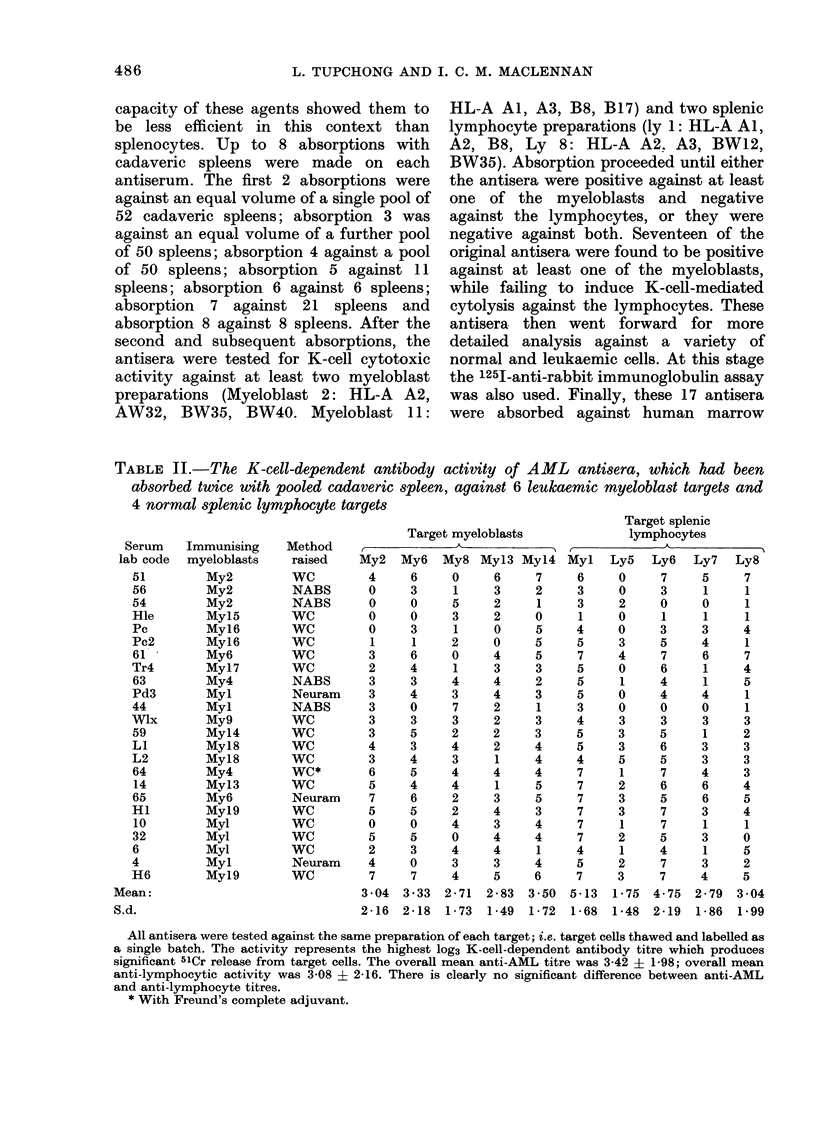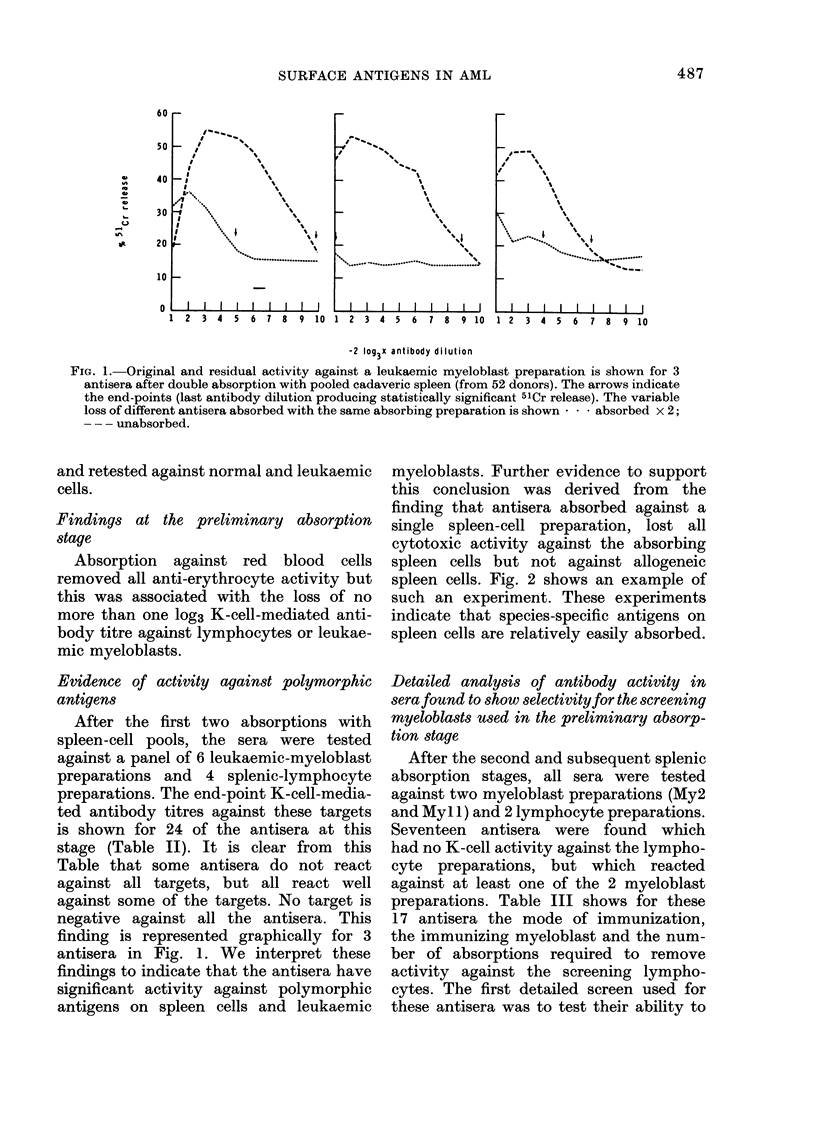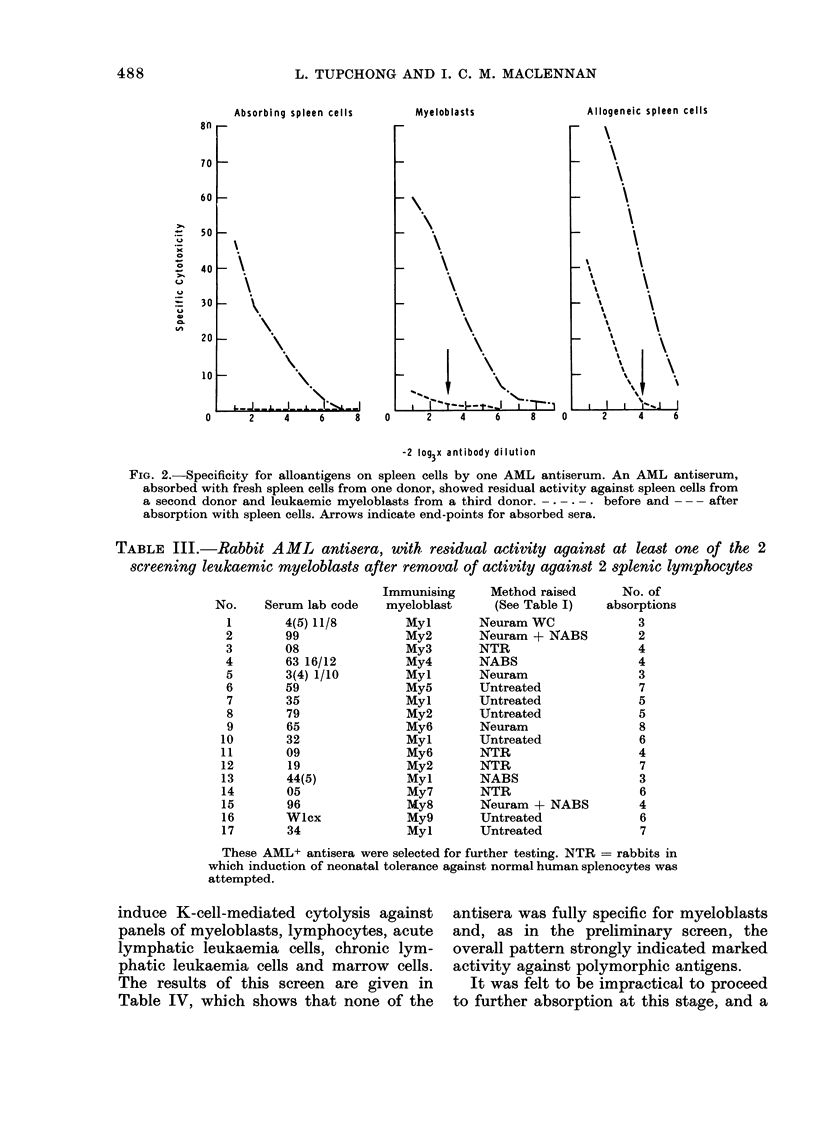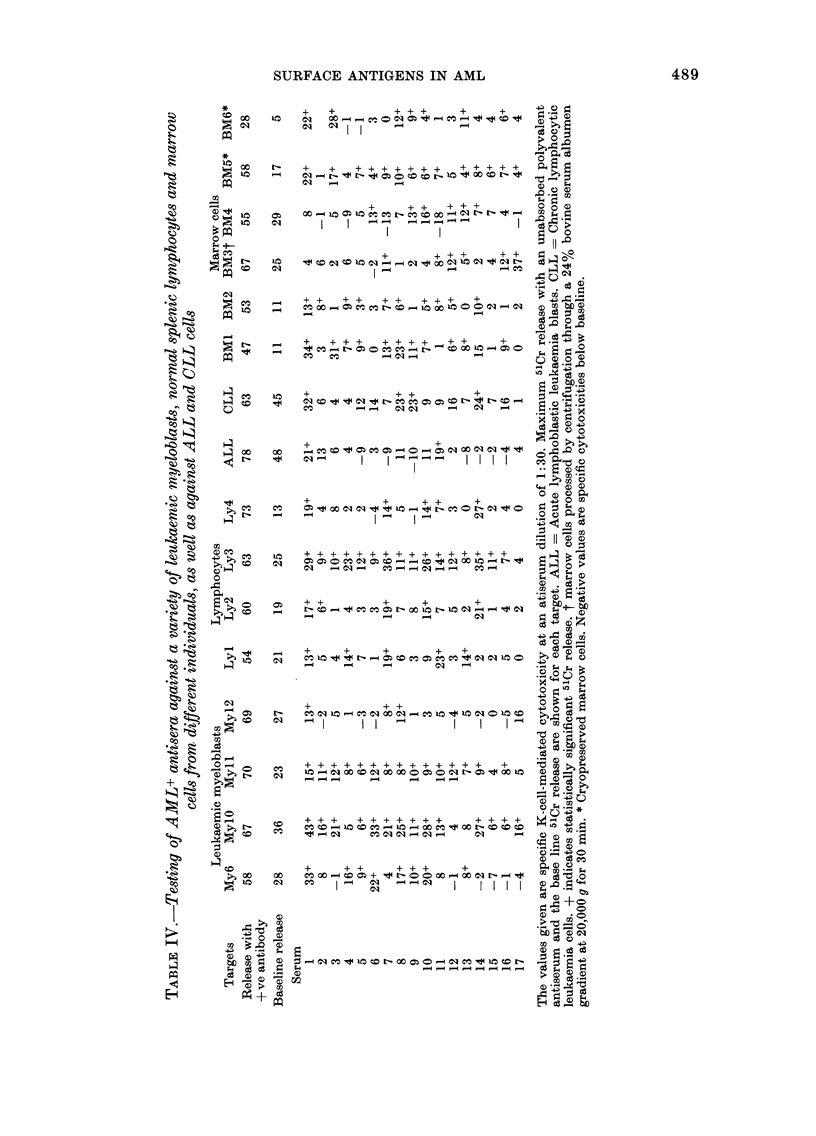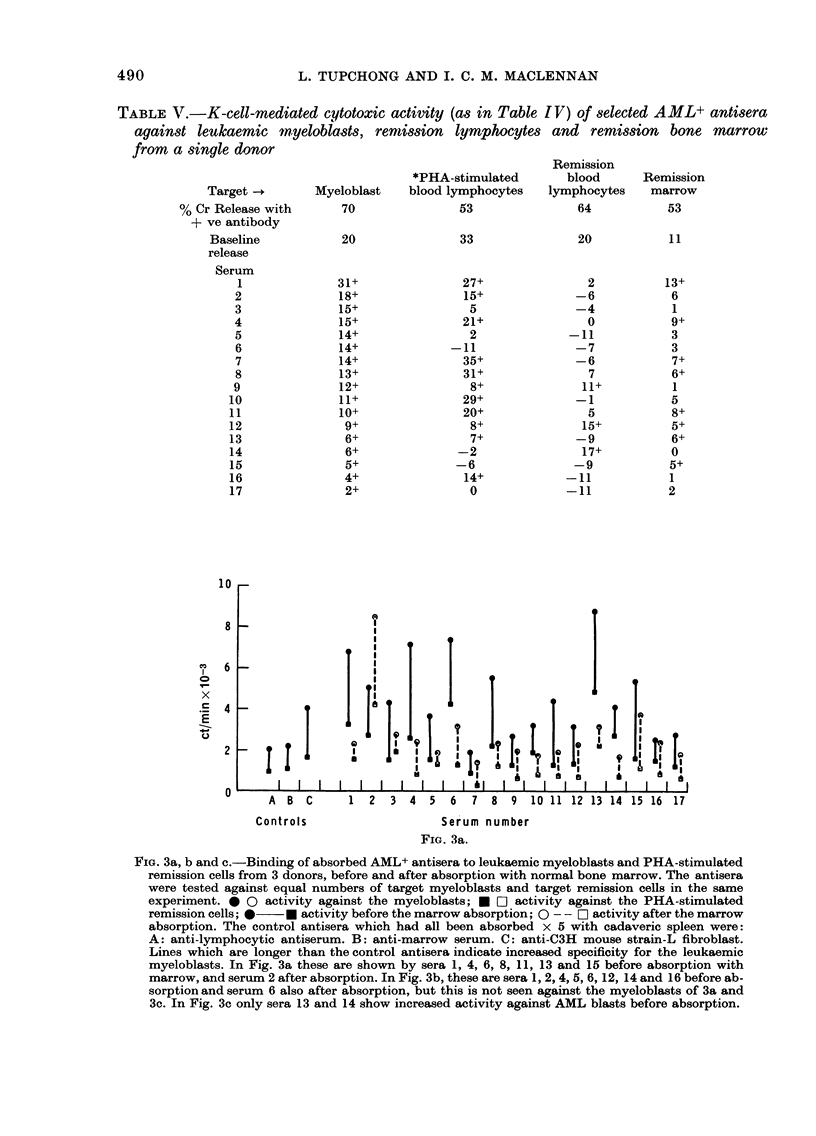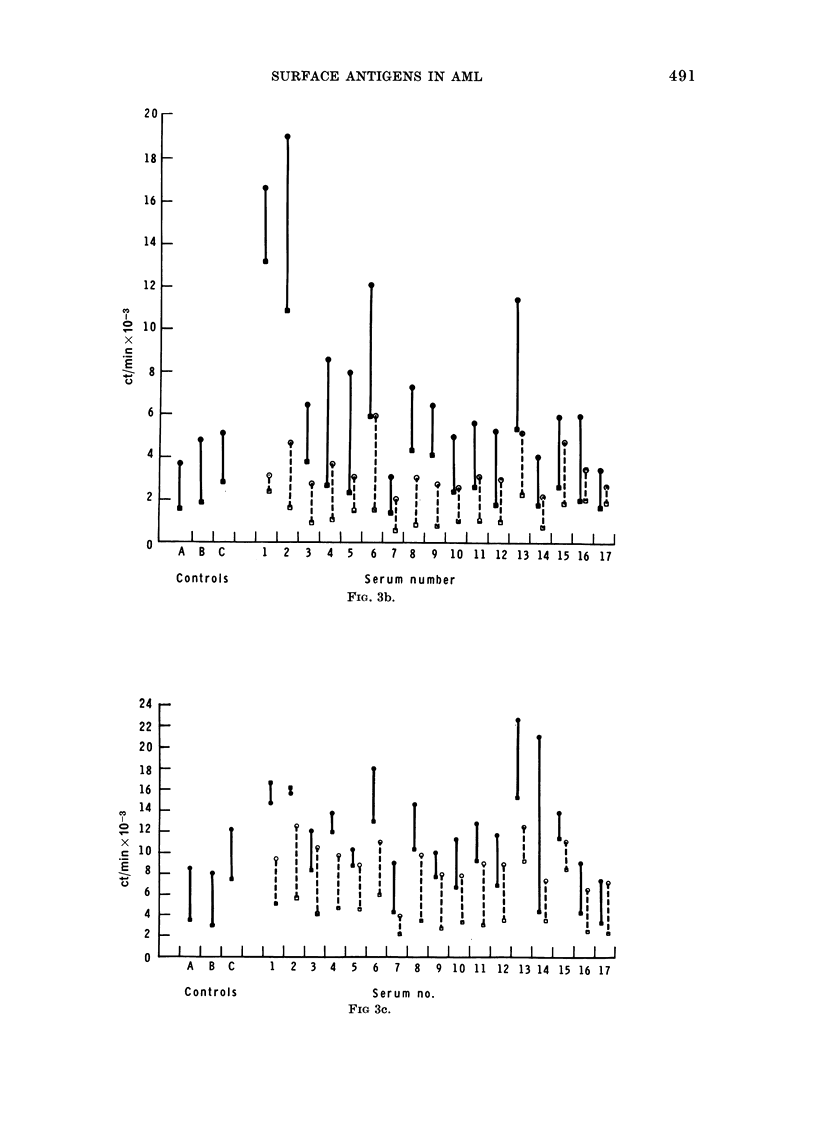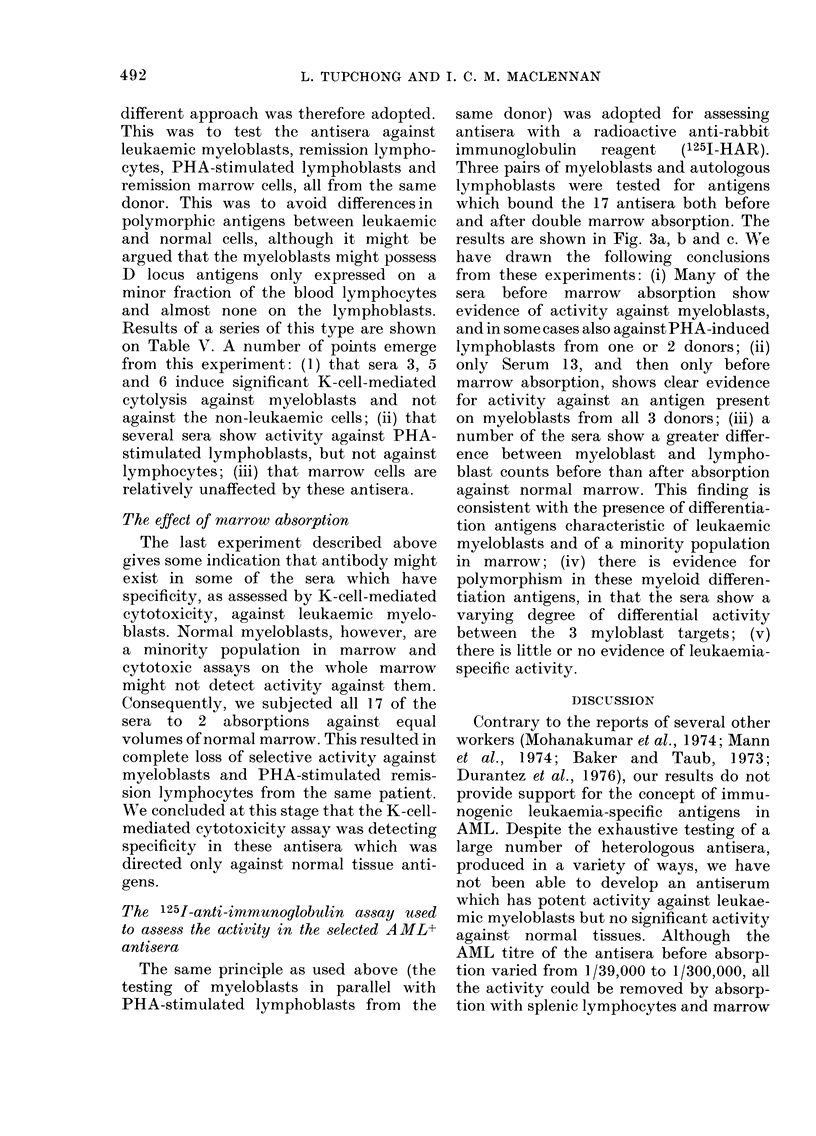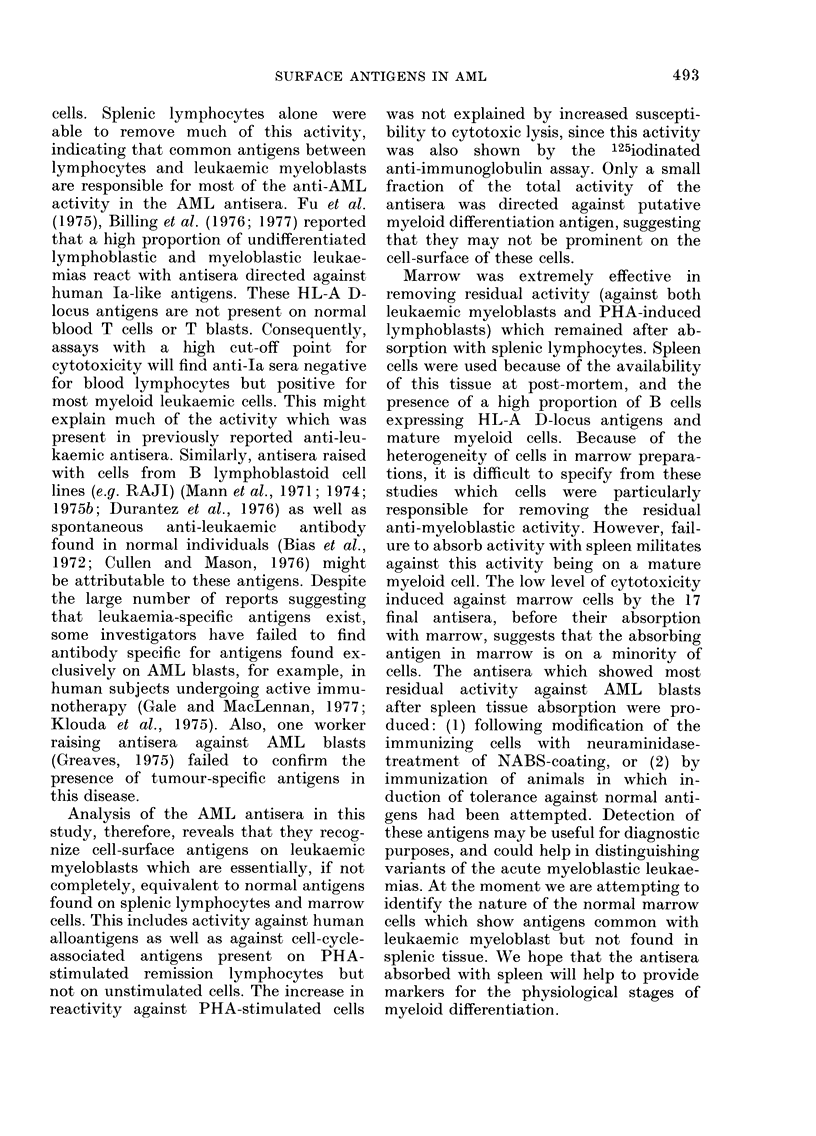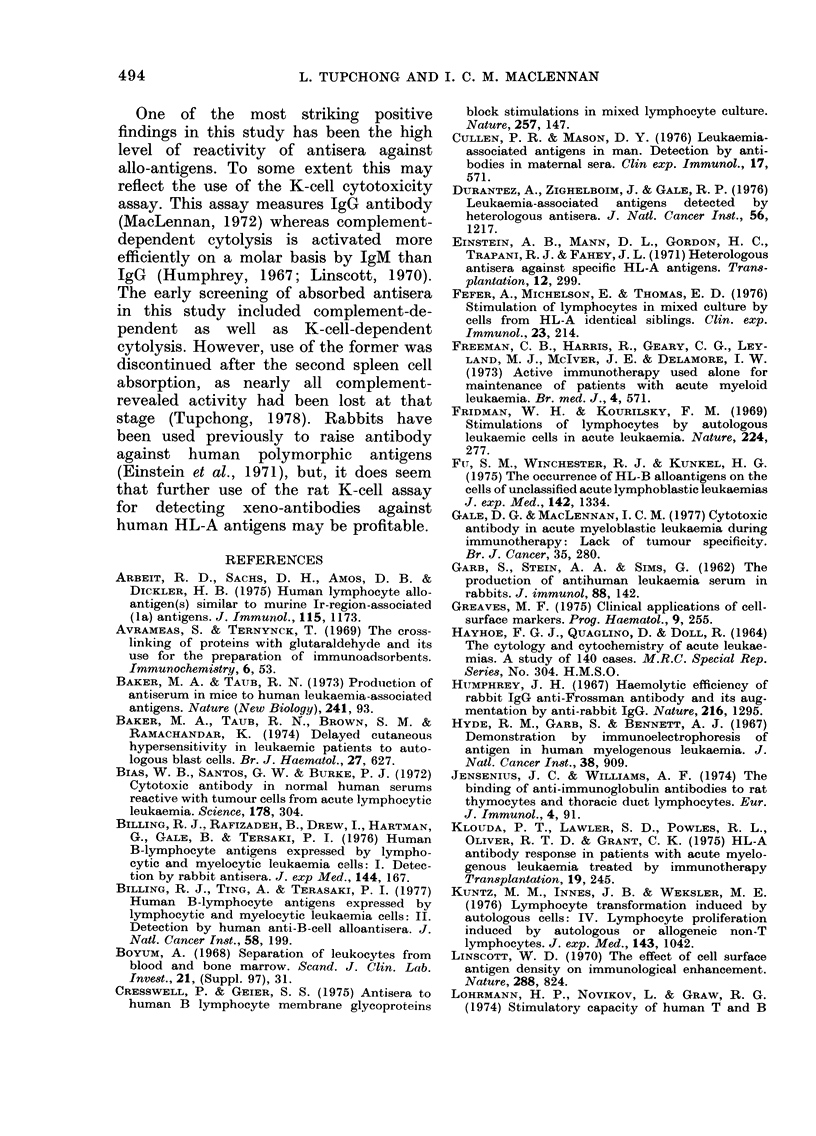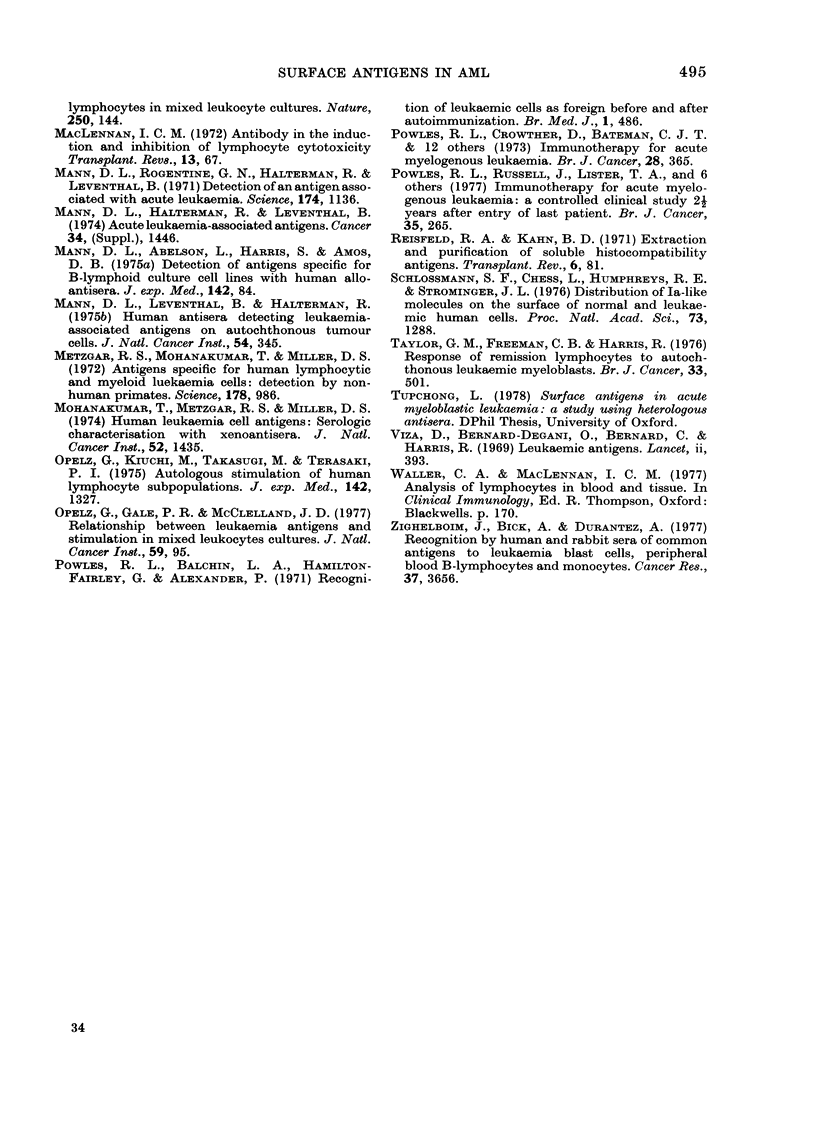# Surface antigens in acute myeloblastic leukaemia: a study using heterologous antisera.

**DOI:** 10.1038/bjc.1978.235

**Published:** 1978-10

**Authors:** L. Tupchong, I. C. MacLennan

## Abstract

This study analyses the activity of 95 antisera raised in rabbits against human leukaemic myeloblasts. A number of different means were used to immunize both normal rabbits and rabbits which had been treated to render them tolerant of normal human splenic leucocytes. Different immunization schedules included the use of different doses of untreated myeloblasts, as well as myeloblasts treated with neuraminidase, antibody against human spleen cells or glutaraldehyde. Analysis of the sera was carried out using two sensitive techniques for detecting cell surface antigens: a radioactive anti-immunoglobulin binding assay using 125I-horse-F(ab')2-anti-rabbit-Fab and a K-cell-mediated cytotoxicity test using rat spleen cells as effectors. (i) The unabsorbed sera showed similar mean titres against leukaemic myeloblasts and normal splenocytes. (ii) Extensive absorption with pooled cadaveric spleen were required to remove antibody against polymorphic antigens. (iii) 17/95 antisera had activity against at least some leukaemic myeloblasts after extensive absorption with cadaveric spleen. (iv) Some of the 17 absorbed sera with selective activity for myeloblasts also reacted against PHA-induced lymphoblasts. (v) Although the 17 absorbed sera showed little or no activity against marrow in the above assays normal human marrow totally absorbed all residual activity in these sera against leukaemic myeloblasts. We conclude that although these sera contain activity against antigens common to leukaemic myeloblasts and a minority population of normal marrow cells, they have no detectable activity against leukaemic-specific antigens.


Br. J. Cancer (1978) 38, 481

### SURFACE ANTIGENS IN ACUTE MYELOBLASTIC LEUKAEMIA: A STUDY USING HETEROLOGOUS ANTISERA

L. TUPCHONG AND I. C. M. MACLENNAN

*From the Nuffield Department of Clinical Medicine, Radcliffe Infirmary, Oxford OX2 6HE*

Received 18 July 1978 Accepted 31 July 1978

**Summary.—This study analyses the activity of 95 antisera raised in rabbits against human leukaemic myeloblasts. A number of different means were used to immunize both normal rabbits and rabbits which had been treated to render them tolerant of normal human splenic leucocytes. Different immunization schedules included the use of different doses of untreated myeloblasts, as well as myeloblasts treated with neuraminidase, antibody against human spleen cells or glutaraldehyde.**

**Analysis of the sera was carried out using two sensitive techniques for detecting cell surface antigens: a radioactive anti-immunoglobulin binding assay using $^{125}$I-horse-F(ab′)$_2$-anti-rabbit-Fab and a K-cell-mediated cytotoxicity test using rat spleen cells as effectors.**

**(i) The unabsorbed sera showed similar mean titres against leukaemic myeloblasts and normal splenocytes.**

**(ii) Extensive absorption with pooled cadaveric spleen were required to remove antibody against polymorphic antigens.**

**(iii) 17/95 antisera had activity against at least some leukaemic myeloblasts after extensive absorption with cadaveric spleen.**

**(iv) Some of the 17 absorbed sera with selective activity for myeloblasts also reacted against PHA-induced lymphoblasts.**

**(v) Although the 17 absorbed sera showed little or no activity against marrow in the above assays normal human marrow totally absorbed all residual activity in these sera against leukaemic myeloblasts.**

**We conclude that although these sera contain activity against antigens common to leukaemic myeloblasts and a minority population of normal marrow cells, they have no detectable activity against leukaemia-specific antigens.**

DESPITE much circumstantial evidence for their presence, the existence of human tumour-specific antigens remains a tentative proposition. Nevertheless, over the years there have been several reports of the production of heterologous antisera specific for leukaemia-associated antigens in acute myeloblastic leukaemia (AML) and other leukaemias (Garb *et al.*, 1962; Hyde *et al.*, 1967; Mann *et al.*, 1971; Mann *et al.*, 1974; Metzgar *et al.*, 1972; Mohanakumar *et al.*, 1974; Baker *et al.*, 1974; Durantez *et al.*, 1976). In addition, stimulation of lymphocyte blastogenesis by leukaemic cells, particularly in the autologous situation (Fridman and Kourilsky, 1969; Viza *et al.*, 1969; Powles *et al.*, 1971; Taylor *et al.*, 1976) and in HL-A-identical siblings (Fefer *et al.*, 1976) has been presented as evidence for the existence of these antigens. These findings have generated much interest in the possibility of exploiting these leukaemia antigens in active immunotherapy to prolong the duration of clinical remission in AML (Powles *et al.*, 1973; Freeman *et al.*, 1973; Powles *et al.*, 1977).

Recently, HL-A D-locus antigens commonly associated with B-cells (Ia antigens) (Arbeit *et al.*, 1975; Mann *et al.*, 1975*a*) have been reported on the surface of a high proportion of acute leukaemia cells (Fu *et al.*, 1975; Schlossmann *et al.*, 1976; Billing *et al.*, 1976, 1977). It is clear that several antisera which were previously reported to be specific for leukaemia are, in fact, directed against these antigens (Billing *et al.*, 1976; Zighelboim *et al.*, 1977). Furthermore, the stimulation of autologous and HL-A-identical peripheral blood lymphocytes by leukaemic cells may be related to the presence of these antigens on leukaemic cells (Opelz *et al.*, 1977). Autologous MLC stimulation by normal lymphocyte subpopulations occurs in analogous conditions with a high T-cell-responding cell population and a B-cell-enriched stimulating population (Lohrmann *et al.*, 1974; Opelz *et al.*, 1975; Kuntz *et al.*, 1976) and antisera against Ia antigens inhibit these reactions (Cresswell and Geier, 1975).

In this report we describe attempts to confirm the presence of leukaemia-specific antigens, by producing rabbit antisera against human leukaemic myeloblasts by a variety of techniques. The specificities of the heterologous antisera produced against AML have been analysed by 2 sensitive techniques, a K-cell-mediated cytotoxicity test and a non-cytotoxic radio-immune anti-immunoglobulin binding technique. The results suggest that the surface antigenic make-up of leukaemic myeloblasts is qualitatively normal. The antigenic determinants present on leukaemic myeloblasts include those which are characteristic for a minority population of normal marrow cells. There is also evidence for antibodies in these AML antisera which are reactive with PHA-stimulated lymphoblasts and which cannot be absorbed out by splenic lymphoid cells. If leukaemia-specific antigens common to most patients with AML exist, they are either non-immunogenic in the animals studied or are below the resolution of the tests used for their detection in this study.

### METHODS AND MATERIALS

*Patients*.—These were adults admitted to the Radcliffe Infirmary, Oxford, which was one of the centres involved in the recent British Medical Research Council's 6th AML Immunotherapy Trial. The diagnosis of acute myeloblastic leukaemia (AML) was made on clinical grounds, and on conventional morphological criteria and cytochemistry with Sudan Black B, PAS and Romanovsky stains (Hayhoe *et al.*, 1964). Patients were selected for study only if they had at least 90% myeloblasts in the peripheral blood.

*Collection and storage of myeloblasts*.—Blood was taken into heparin or acid-citrate dextrose from each patient at clinical presentation before chemotherapy was instituted. Myeloblasts were separated from peripheral blood and marrow aspirates by Ficoll-Triosil gradient centrifugation (Boyum, 1968) or after the sedimentation of blood under 1 *g* and the removal of the leucocyte-rich plasma. The cells were washed twice in pre-heated normal saline (37°C) to remove free plasma and passively carried proteins and then resuspended in RPMI 1640 (Gibco-Biocult, Paisley) which was supplemented with 10% foetal calf serum, 1% non-essential amino acids, 2 mM fresh glutamine, penicillin and streptomycin. Dimethyl sulphoxide was added to the cell suspension to a final concentration of 10%. The cells were then rapidly dispensed into polycarbonate ampoules (Sterilin, Surrey) and then cooled at 1–2°C/min until the temperature had been lowered to $\sim -60$°C. The ampoules were then stored over liquid $N_2$.

The cells were thawed rapidly in a 37°C waterbath and diluted gradually with pre-warmed normal saline + 10% FBS before use. They were washed twice with normal saline. The viability of intact cells after thawing, as assessed by trypan-blue dye exclusion and phase microscopy was 70–95%.

*Splenic lymphocytes*.—Viable cells were obtained form normal spleens from patients undergoing operation for acute splenic rupture and stored by controlled-rate freezing.

*Cadaveric spleens*.—Macroscopically normal cadaveric spleens were obtained within 48 h of death from individuals who were free of malignancy and overt infection, and were stored at $-20$°C until used. The validity of using spleens frozen at $-20$°C has been extensively assessed with traumatic spleens used fresh and after being processed as cadaveric spleens. The absorptive capacity of material prepared by these two techniques could not be distinguished (Tupchong, 1978). Before use, spleens were thawed at 37°C, homogenized in a moule (Mouli-baby grinder) and thoroughly washed with phosphate-buffered saline.

*Marrow.*—Fresh marrow cells were obtained from ribs removed from patients undergoing thoracotomy for non-malignant conditions. As target cells, they were used fresh or after storage in liquid $N_2$ after controlled-rate freezing. When used for absorption purposes, they were lightly fixed with glutaraldehyde (0·25%) (Avrameas and Ternynck, 1969) and stored at 4°C in 0·1% sodium azide.

*Remission lymphocytes.*—Lymphocytes were isolated from defibrinated peripheral blood of patients during clinical remission by centrifugation (400 *g*) over a Ficoll-Triosil gradient (sp.gr. 1·077) for 15 min at 22°C. The interface contains 95% mononuclear cells, mainly lymphocytes.

*Production of antisera.*—Antisera were raised in rabbits from a variety of breeds, including Blue, Chinchilla, Grey and New Zealand White. A variety of immunological procedures were used in attempts to improve the specificity for tumour antigens (Table I):

(a) Untreated whole cells: Animals were injected 2–3 times i.p. with washed fresh or cryopreserved myeloblasts. $10^8$ cells were normally used for each injection, but in some rabbits either $10^6$ or $10^9$ cells were used in the primary immunization. Injections were spaced at 2-week intervals.

(b) Normal - antigen - blocking - serum (NABS)-coated cells: NABS consisted of a heat-inactivated pooled anti-lymphocytic and anti-marrow serum from 9 rabbits which had been immunized with human splenic lymphocytes or fresh human marrow cells. Each rabbit contributing to the pool received cells from only one individual. Two injections of $10^8$ cells were given 14 days apart. The rabbits were bled on Day 21. The dose of NABS required to coat fully $10^8$ myeloblasts was calculated from a complement mediated titration curve against $2 \times 10^4$ of the relevant myeloblasts. Cryopreserved myeloblasts were washed and then resuspended in $\times$ 20 the volume calculated. After 30 min, the cells were injected i.p. together with the NABS. This procedure was repeated for each immunization. Injections of $10^8$ NABS-treated cells were given i.p. on Days 1 and 14 and the animals were bled on Day 21–25.

(c) Neuraminidase-treated cells: Washed myeloblasts were resuspended in normal saline at a concentration of $3 \times 10^7$ cells/ml. *Vibrio cholerae* neuraminidase (Behringwerke AG Batch 1233 B, 25 or 50 i.u./$3 \times 10^7$ cells) was added and the mixture was incubated for 30 min at 37°C. The cells were then washed twice in cold PBS, enumerated and injected immediately.

(d) Gluteraldehyde-fixed cells: 0·25% glutaraldehyde (BDH) was added to washed myeloblasts (at a concentration of $10^7$/ml) in normal saline. After 5 min at room temperature, the cells were washed $\times$ 3 in normal saline.

*Induction of tolerance of human spleen cells.*—(1) Neonatal injection with human cells: Neonatal rabbits were injected i.p. within 3 h of birth with $1–2 \times 10^8$ human spleen cells (carefully cryopreserved from ruptured spleens). Injections were given daily for 12 days and then every 2 days for a week. In some rabbits this was followed by twice-weekly injections until 4 weeks after birth. When rabbits were test-bled at one month of age, all had some antibody against splenocytes, and the degree of tolerance achieved was in some doubt. In further attempts to induce tolerance in these animals, cyclophosphamide (Endoxana, Wellcome, Kent) (100 mg/kg) was given i.p. together with $10^8$ splenic lymphocytes. In several rabbits (see Table I), the cyclophosphamide was repeated either alone or with splenic lymphocytes every 2 weeks. (ii) Neonatal injection with soluble human antigen: Soluble human antigen was prepared by 3M KCl extraction (Reisfeld and Kahn, 1971) from $5 \times 10^9$ normal splenic lymphocytes. The preparation was ultracentrifuged at 160,000 *g* for 90 min and the supernatant was tested for its ability to inhibit the action of an anti-lymphocytic serum against the original splenic lymphocytes as target cells. Neonatal rabbits were injected i.p. daily with 10 mg of the soluble extract for 10 days. They were test bled at one month and challenged with cyclophosphamide and normal splenic lymphocytes. At this stage, none of the rabbits which were injected with soluble material had antibody against normal splenocytes. (iii) Attempts to induce tolerance in adult rabbits: Adult rabbits were injected i.p. at 3 months of age with cyclophosphamide (100 mg/kg) together with 1–2 $\times 10^8$ splenic lymphocytes.

Irrespective of the method used to induce tolerance, all rabbits were injected 14 days after the last injection of cyclophosphamide with $10^8$ leukaemic myeloblasts and this was repeated thrice at 14-day intervals. In some animals, the immunizing myeloblasts were coated with NABS prior to injection.

*Control antisera.*—The following immune-rabbit antisera were used as controls in the absorption procedures: an anti-human-lymphocyte serum, an anti-human-marrow serum, an anti-C3H mouse L-strain-fibroblast serum and an anti-C3H mouse benzopyrene-induced-sarcoma serum.

*Absorption of antisera.*—Washed human splenic homogenate and bone marrow cells were used to remove activity against normal tissue. The absorbing tissue was packed at 2200 *g* for 10 min and an equal volume of antiserum was added. The mixture was resuspended and allowed to react for 1 h at 4 °C with frequent inversion. The antiserum was removed after centrifugation at 20,000 *g* for 10 min. No antiserum was absorbed more than twice with the same preparation of absorbing material.

*Phytohaemagglutinin (PHA) stimulation of lymphocytes.*—Lymphocytes were suspended in RPMI-1640-FBS at a concentration of $2 \times 10^6$ cells/ml and dispensed in 500 $\mu$l aliquots into sterile 76 $\times$ 10 mm round-bottomed plastic tubes (Sterilin, Middlesex). Five hundred $\mu$l of PHA (Wellcome Reagents, Beckenham, Kent, Batch no. K.0979) diluted 6 parts in 1,000 with RPMI-FBS was added to each tube. Unstimulated control tubes received 500 $\mu$l MEM only. All tubes were then tightly capped and incubated at 37 °C for 72 h. The stimulated and unstimulated control tubes were each pulsed at the end of 68 h with 1 $\mu$Ci (50 $\mu$l) of [$3_H$]TdR (Radiochemical Centre, Amersham). The unpulsed PHA-stimulated cells were washed in medium and used as target cells for testing the AML antisera. The degree of stimulation was determined by [$3_H$]TdR uptake of an aliquot of the stimulated cells using the technique of Waller and MacLennan (1977). The ratio of counts in stimulated and unstimulated tubes (stimulation index) varied between 12·6 and 20·0.

*$^{51}$Cr- labelling of cells.*—Between $5 \times 10^6$ and $10^7$ myeloblasts in 0·3 ml FBS were labelled with 100 $\mu$Ci sodium chromate (Radiochemical Centre, Amersham, sp. act. 100–400 mCi/$\mu$gCr) for 2 h at 37 °C. For labelling lymphocytes, 200–300 $\mu$Ci of $^{51}$Cr was used. After labelling, the cells were washed in medium through a gradient of FBS carefully layered at the bottom of the container.

*The assays*

*K-cell-mediated cytotoxicity.*—Heat-inactivated antisera were serially diluted 1:3 in 100 $\mu$l BME in Cooke microtitre plates. Fifty microlitres of rat spleen cells from Lewis rats (RSC, $6 \times 10^7$ cells/ml) were added, followed by 50 $\mu$l $^{51}$Cr-labelled target cells ($4 \times 10^5$ cells/ml).

Controls consisted of (i) target cells, RSC and medium without antibody (baseline release—6 readings); (ii) target cells, RSC and a known positive antiserum, diluted 1/20, which was at the plateau of cytotoxicity.

The plates were sealed with non-toxic adherent plastic film and incubated for 6 h at 37 °C. They were then centrifuged at 200 *g* for 5 min and 150 $\mu$l of the supernatant from each well was transferred to small plastic tubes for counting in an automatic Wallac gamma-counter. Six tubes were also added which contained the total activity added to each well. The counts were processed according to a programme designed to calculate percentage actual $^{51}$Cr release, means, standard deviation and specific cytotoxicity values. The following formulae were incorporated into the programme: percentage actual $^{51}$Cr release:

$$\frac{(\text{Supernatant aliquot ct/min} \times 1{\cdot}33) - \text{background}}{\text{total counts} - \text{background}} \times 100$$

Specific cytotoxicity:

$$\frac{(\%\ \text{release with test antibody} - \%\ \text{baseline release})}{\%\ \text{release in positive control} - \%\text{baseline release}} \times 100$$

Mean duplicate assay values (percent actual $^{51}$Cr release) were compared to mean baseline values (6 readings) for each dilution of the antiserum and were considered significantly different if:

$$\text{mean assay value} - 2\ \text{s.e.}_1 > \text{mean baseline value} + 2\ \text{s.e.}_2$$

where: $\text{s.e.}_1$ = standard error of mean of assay readings
$\text{s.e.}_2$ = standard error of mean of baseline readings.

The last dilution of the antiserum producing significant $^{51}$Cr release above baseline ($P < 0{\cdot}05$) was taken as the end-point titre of the antiserum. We are grateful to Dr G. T. Warner for preparing the computer programme.

*$^{125}$Iodinated horse anti-rabbit ($^{125}$I-HAR) binding assay.*—A horse anti-rabbit serum (generous gift of Dr A. F. Williams, Department of Biochemistry, Oxford) was purified by elution from a Sepharose 4B immunoabsorbent column (rabbit IgG), followed by pepsin degradation. Pepsin (Sigma) was added at 4 mg/100 mg protein for 20 h at 37°C in 0·1M sodium acetate buffer, pH 4·5. The mixture was then passaged through a Sephadex G200 filtration column. The $F(ab')_2$ peak was taken and iodinated using a low dose of chloramine T (BDH) (10–20 μg per 25 μg protein) using the technique of Jensenius and Williams (1974).

The assay was performed in round-bottomed Cooke microtitre plates (Sterilin). To standard 3-fold dilutions of heat-inactivated antiserum in MEM was added live target cells at $\sim 5 \times 10^5$ cells per well. After preincubation of the antibody and target cells for 1 h at 0–4°C, the cells were washed × 3 with PBS + 1% BSA and 0·02% azide. The radio-labelled HAR antibody was then added to the pelleted cells at 300,000–400,000 ct/min. The cells were resuspended and left for a further 60 min, after which they were washed × 3. The final pellet was dissolved in 100 μl NaOH (0·1M) and transferred to small plastic tubes for gamma-counting.

### RESULTS

*General plan for assessing specificity of antisera raised against leukaemic myeloblasts*

Antisera were tested from 95 different rabbits each immunized with leukaemic myeloblasts from a single patient. Myeloblasts from 19 patients were used for immunization. Several different immunization schedules were used, as well as a variety of techniques to reduce the level of antibody against normal tissue antigens. These immunization schedules are summarized in Table I.

TABLE I.—*Immunization schedules used to raise rabbit AML antisera (see Methods)*

| Schedule | No. of antisera |
|---|---|
| Untreated whole cells* (wc) | |
| $10^6$; $10^8$ | 5 |
| $10^7$; $10^8$ | 5 |
| $10^8$; $10^8$; $10^8$ | 31 |
| $10^9$; $10^8$ | 4 |
| Normal-antigen-blocking serum (NABS)—coated cells | 6 |
| Neuraminidase-treated cells (Neuram) | |
| 25 i.u./3 × $10^7$ cells/ml + NABS | 5 |
| 50 i.u./3 × $10^7$ cells/ml | 4 |
| Glutaraldehyde-fixed cells | 4 |
| After tolerizing procedures: | |
| neonatal injection with soluble lymphocyte-antigen | |
| + Cy† (× 2) with splenic cells | 9 |
| neonatal injection with whole cells | 1 |
| + Cy† and splenic cells | 9 |
| + Cy† (× 2) and splenic cells (× 2) | 2 |
| + Cy† (× 2) and splenic cells | 1 |
| + Cy† and splenic cells (× 2) | 3 |
| Adult tolerization: | |
| Cy† and splenic cells | 1 |
| Cy† and splenic cells and NABS | 5 |

* Immunizing doses are indicated in 2 or 3 successive doses as shown.
† Cy = cyclophosphamide at 100 mg/kg/dose.

Before any absorption, the antisera were tested for their capacity to induce cytolysis by rat spleen K-cells against a standard test leukaemic-myeloblast preparation and a test normal splenic-lymphocyte preparation. All antisera had strong activity against both, with the mean end-point titre against the myeloblasts ± 1 s.d. being $3^{8{\cdot}12 \pm 1{\cdot}24}$ and against the splenic lymphocytes $3^{7{\cdot}58 \pm 1{\cdot}96}$.

The antisera were next passed through a series of absorptions against pooled cadaveric human spleen cells. Some sera were also absorbed against erythrocytes and platelets, but analysis of the absorptive capacity of these agents showed them to be less efficient in this context than splenocytes. Up to 8 absorptions with cadaveric spleens were made on each antiserum. The first 2 absorptions were against an equal volume of a single pool of 52 cadaveric spleens; absorption 3 was against an equal volume of a further pool of 50 spleens; absorption 4 against a pool of 50 spleens; absorption 5 against 11 spleens; absorption 6 against 6 spleens; absorption 7 against 21 spleens and absorption 8 against 8 spleens. After the second and subsequent absorptions, the antisera were tested for K-cell cytotoxic activity against at least two myeloblast preparations (Myeloblast 2: HL-A A2, AW32, BW35, BW40. Myeloblast 11: HL-A A1, A3, B8, B17) and two splenic lymphocyte preparations (ly 1: HL-A A1, A2, B8, Ly 8: HL-A A2, A3, BW12, BW35). Absorption proceeded until either the antisera were positive against at least one of the myeloblasts and negative against the lymphocytes, or they were negative against both. Seventeen of the original antisera were found to be positive against at least one of the myeloblasts, while failing to induce K-cell-mediated cytolysis against the lymphocytes. These antisera then went forward for more detailed analysis against a variety of normal and leukaemic cells. At this stage the $^{125}$I-anti-rabbit immunoglobulin assay was also used. Finally, these 17 antisera were absorbed against human marrow

TABLE II.—*The K-cell-dependent antibody activity of AML antisera, which had been absorbed twice with pooled cadaveric spleen, against* 6 *leukaemic myeloblast targets and* 4 *normal splenic lymphocyte targets*

| Serum lab code | Immunising myeloblasts | Method raised | Target myeloblasts My2 | My6 | My8 | My13 | My14 | Target splenic lymphocytes My1 | Ly5 | Ly6 | Ly7 | Ly8 |
|---|---|---|---|---|---|---|---|---|---|---|---|---|
| 51 | My2 | WC | 4 | 6 | 0 | 6 | 7 | 6 | 0 | 7 | 5 | 7 |
| 56 | My2 | NABS | 0 | 3 | 1 | 3 | 2 | 3 | 0 | 3 | 1 | 1 |
| 54 | My2 | NABS | 0 | 0 | 5 | 2 | 1 | 3 | 2 | 0 | 0 | 1 |
| Hle | My15 | WC | 0 | 0 | 3 | 2 | 0 | 1 | 0 | 1 | 1 | 1 |
| Pc | My16 | WC | 0 | 3 | 1 | 0 | 5 | 4 | 0 | 3 | 3 | 4 |
| Pc2 | My16 | WC | 1 | 1 | 2 | 0 | 5 | 5 | 3 | 5 | 4 | 1 |
| 61 | My6 | WC | 3 | 6 | 0 | 4 | 5 | 7 | 4 | 7 | 6 | 7 |
| Tr4 | My17 | WC | 2 | 4 | 1 | 3 | 3 | 5 | 0 | 6 | 1 | 4 |
| 63 | My4 | NABS | 3 | 3 | 4 | 4 | 2 | 5 | 1 | 4 | 1 | 5 |
| Pd3 | My1 | Neuram | 3 | 4 | 3 | 4 | 3 | 5 | 0 | 4 | 4 | 1 |
| 44 | My1 | NABS | 3 | 0 | 7 | 2 | 1 | 3 | 0 | 0 | 0 | 1 |
| Wlx | My9 | WC | 3 | 3 | 3 | 2 | 3 | 4 | 3 | 3 | 3 | 3 |
| 59 | My14 | WC | 3 | 5 | 2 | 2 | 3 | 5 | 3 | 5 | 1 | 2 |
| L1 | My18 | WC | 4 | 3 | 4 | 2 | 4 | 5 | 3 | 6 | 3 | 3 |
| L2 | My18 | WC | 3 | 4 | 3 | 1 | 4 | 4 | 5 | 5 | 3 | 3 |
| 64 | My4 | WC* | 6 | 5 | 4 | 4 | 4 | 7 | 1 | 7 | 4 | 3 |
| 14 | My13 | WC | 5 | 4 | 4 | 1 | 5 | 7 | 2 | 6 | 6 | 4 |
| 65 | My6 | Neuram | 7 | 6 | 2 | 3 | 5 | 7 | 3 | 5 | 6 | 5 |
| H1 | My19 | WC | 5 | 5 | 2 | 4 | 3 | 7 | 3 | 7 | 3 | 4 |
| 10 | My1 | WC | 0 | 0 | 4 | 3 | 4 | 7 | 1 | 7 | 1 | 1 |
| 32 | My1 | WC | 5 | 5 | 0 | 4 | 4 | 7 | 2 | 5 | 3 | 0 |
| 6 | My1 | WC | 2 | 3 | 4 | 4 | 1 | 4 | 1 | 4 | 1 | 5 |
| 4 | My1 | Neuram | 4 | 0 | 3 | 3 | 4 | 5 | 2 | 7 | 3 | 2 |
| H6 | My19 | WC | 7 | 7 | 4 | 5 | 6 | 7 | 3 | 7 | 4 | 5 |
| Mean: | | | 3·04 | 3·33 | 2·71 | 2·83 | 3·50 | 5·13 | 1·75 | 4·75 | 2·79 | 3·04 |
| S.d. | | | 2·16 | 2·18 | 1·73 | 1·49 | 1·72 | 1·68 | 1·48 | 2·19 | 1·86 | 1·99 |

All antisera were tested against the same preparation of each target; *i.e.* target cells thawed and labelled as a single batch. The activity represents the highest $\log_3$ K-cell-dependent antibody titre which produces significant $^{51}$Cr release from target cells. The overall mean anti-AML titre was 3·42 ± 1·98; overall mean anti-lymphocytic activity was 3·08 ± 2·16. There is clearly no significant difference between anti-AML and anti-lymphocyte titres.

* With Freund's complete adjuvant.

FIG. 1.—Original and residual activity against a leukaemic myeloblast preparation is shown for 3 antisera after double absorption with pooled cadaveric spleen (from 52 donors). The arrows indicate the end-points (last antibody dilution producing statistically significant $^{51}$Cr release). The variable loss of different antisera absorbed with the same absorbing preparation is shown · · · absorbed × 2; – – – unabsorbed.

and retested against normal and leukaemic cells.

*Findings at the preliminary absorption stage*

Absorption against red blood cells removed all anti-erythrocyte activity but this was associated with the loss of no more than one $\log_3$ K-cell-mediated antibody titre against lymphocytes or leukaemic myeloblasts.

*Evidence of activity against polymorphic antigens*

After the first two absorptions with spleen-cell pools, the sera were tested against a panel of 6 leukaemic-myeloblast preparations and 4 splenic-lymphocyte preparations. The end-point K-cell-mediated antibody titres against these targets is shown for 24 of the antisera at this stage (Table II). It is clear from this Table that some antisera do not react against all targets, but all react well against some of the targets. No target is negative against all the antisera. This finding is represented graphically for 3 antisera in Fig. 1. We interpret these findings to indicate that the antisera have significant activity against polymorphic antigens on spleen cells and leukaemic myeloblasts. Further evidence to support this conclusion was derived from the finding that antisera absorbed against a single spleen-cell preparation, lost all cytotoxic activity against the absorbing spleen cells but not against allogeneic spleen cells. Fig. 2 shows an example of such an experiment. These experiments indicate that species-specific antigens on spleen cells are relatively easily absorbed.

*Detailed analysis of antibody activity in sera found to show selectivity for the screening myeloblasts used in the preliminary absorption stage*

After the second and subsequent splenic absorption stages, all sera were tested against two myeloblast preparations (My2 and My11) and 2 lymphocyte preparations. Seventeen antisera were found which had no K-cell activity against the lymphocyte preparations, but which reacted against at least one of the 2 myeloblast preparations. Table III shows for these 17 antisera the mode of immunization, the immunizing myeloblast and the number of absorptions required to remove activity against the screening lymphocytes. The first detailed screen used for these antisera was to test their ability to

FIG. 2.—Specificity for alloantigens on spleen cells by one AML antiserum. An AML antiserum, absorbed with fresh spleen cells from one donor, showed residual activity against spleen cells from a second donor and leukaemic myeloblasts from a third donor. — . — . — . before and — — — after absorption with spleen cells. Arrows indicate end-points for absorbed sera.

TABLE III.—*Rabbit AML antisera, with residual activity against at least one of the* 2 *screening leukaemic myeloblasts after removal of activity against* 2 *splenic lymphocytes*

| No. | Serum lab code | Immunising myeloblast | Method raised (See Table I) | No. of absorptions |
|---|---|---|---|---|
| 1 | 4(5) 11/8 | Myl | Neuram WC | 3 |
| 2 | 99 | My2 | Neuram + NABS | 2 |
| 3 | 08 | My3 | NTR | 4 |
| 4 | 63 16/12 | My4 | NABS | 4 |
| 5 | 3(4) 1/10 | Myl | Neuram | 3 |
| 6 | 59 | My5 | Untreated | 7 |
| 7 | 35 | Myl | Untreated | 5 |
| 8 | 79 | My2 | Untreated | 5 |
| 9 | 65 | My6 | Neuram | 8 |
| 10 | 32 | Myl | Untreated | 6 |
| 11 | 09 | My6 | NTR | 4 |
| 12 | 19 | My2 | NTR | 7 |
| 13 | 44(5) | Myl | NABS | 3 |
| 14 | 05 | My7 | NTR | 6 |
| 15 | 96 | My8 | Neuram + NABS | 4 |
| 16 | Wlcx | My9 | Untreated | 6 |
| 17 | 34 | Myl | Untreated | 7 |

These AML$^+$ antisera were selected for further testing. NTR = rabbits in which induction of neonatal tolerance against normal human splenocytes was attempted.

induce K-cell-mediated cytolysis against panels of myeloblasts, lymphocytes, acute lymphatic leukaemia cells, chronic lymphatic leukaemia cells and marrow cells. The results of this screen are given in Table IV, which shows that none of the antisera was fully specific for myeloblasts and, as in the preliminary screen, the overall pattern strongly indicated marked activity against polymorphic antigens.

It was felt to be impractical to proceed to further absorption at this stage, and a

TABLE IV.—*Testing of AML+ antisera against a variety of leukaemic myeloblasts, normal splenic lymphocytes and marrow cells from different individuals, as well as against ALL and CLL cells*

| | Leukaemic myeloblasts | | | | Lymphocytes | | | | | | Marrow cells | | | | | |
|---|---|---|---|---|---|---|---|---|---|---|---|---|---|---|---|---|
| Targets | My6 | My10 | My11 | My12 | Ly1 | Ly2 | Ly3 | Ly4 | ALL | CLL | BM1 | BM2 | BM3† | BM4 | BM5* | BM6* |
| Release with +ve antibody | 58 | 67 | 70 | 69 | 54 | 60 | 63 | 73 | 78 | 63 | 47 | 53 | 67 | 55 | 58 | 28 |
| Baseline release | 28 | 36 | 23 | 27 | 21 | 19 | 25 | 13 | 48 | 45 | 11 | 11 | 25 | 29 | 17 | 5 |
| Serum | | | | | | | | | | | | | | | | |
| 1 | 33+ | 43+ | 15+ | 13+ | 13+ | 17+ | 29+ | 19+ | 21+ | 32+ | 34+ | 13+ | 4 | 8 | 22+ | 22+ |
| 2 | 8 | 16+ | 11+ | −2 | 5 | 6+ | 9+ | 4 | 13 | 6 | 3 | 8+ | 6 | −1 | 1 | |
| 3 | −1 | 21+ | 12+ | 5 | 4 | 1 | 10+ | 8 | 6 | 4 | 31+ | 1 | 2 | 5 | 17+ | 28+ |
| 4 | 16+ | 5 | 8+ | 1 | 14+ | 4 | 23+ | 2 | 4 | 4 | 7+ | 9+ | 6 | −9 | 4 | −1 |
| 5 | 9+ | 6+ | 6+ | −3 | 7 | 3 | 12+ | 2 | −9 | 12 | 9+ | 3+ | 5 | 5 | 7+ | −1 |
| 6 | 22+ | 33+ | 12+ | −2 | 1 | 3 | 9+ | −4 | 3 | 14 | 0 | 3 | −2 | 13+ | 4+ | 3 |
| 7 | 4 | 21+ | 8+ | 8+ | 19+ | 19+ | 36+ | 14+ | −9 | 7 | 13+ | 7+ | 11+ | −13 | 9+ | 0 |
| 8 | 17+ | 25+ | 8+ | 12+ | 6 | 7 | 11+ | 5 | 11 | 23+ | 23+ | 6+ | 1 | 7 | 10+ | 12+ |
| 9 | 10+ | 11+ | 10+ | 1 | 3 | 8 | 11+ | −1 | −10 | 23+ | 11+ | 1 | 2 | 13+ | 6+ | 9+ |
| 10 | 20+ | 28+ | 9+ | 3 | 9 | 15+ | 26+ | 14+ | 11 | 9 | 7+ | 5+ | 4 | 16+ | 6+ | 4+ |
| 11 | 8 | 13+ | 10+ | 5 | 23+ | 7 | 14+ | 7+ | 19+ | 9 | 1 | 8+ | 8+ | −18 | 7+ | 1 |
| 12 | −1 | 4 | 12+ | −4 | 3 | 5 | 12+ | 3 | 2 | 16 | 6+ | 5+ | 12+ | 11+ | 5 | 3 |
| 13 | 8+ | 8 | 7+ | 5 | 14+ | 2 | 8+ | 0 | −8 | 7 | 8+ | 0 | 5+ | 12+ | 4+ | 11+ |
| 14 | −2 | 27+ | 9+ | −2 | 2 | 21+ | 35+ | 27+ | −2 | 24+ | 15 | 10+ | 2 | 7+ | 8+ | 4 |
| 15 | −7 | 6+ | 4 | 0 | 2 | 1 | 11+ | 2 | −2 | 7 | 1 | 2 | 4 | 7 | 6+ | 4 |
| 16 | −1 | 6+ | 8+ | −5 | 5 | 4 | 7+ | 4 | −4 | 16 | 9+ | 1 | 12+ | 4 | 7+ | 6+ |
| 17 | −4 | 16+ | 5 | 16 | 0 | 2 | 4 | 0 | 4 | 1 | 0 | 2 | 37+ | −1 | 4+ | 4 |

The values given are specific K-cell-mediated cytotoxicity at an atiserum dilution of 1:30. Maximum $^{51}Cr$ release with an unabsorbed polyvalent antiserum and the base line $^{51}Cr$ release are shown for each target. ALL = Acute lymphoblastic leukaemia blasts. CLL = Chronic lymphocytic leukaemia cells. + indicates statistically significant $^{51}Cr$ release. † marrow cells processed by centrifugation through a 24% bovine serum albumen gradient at 20,000 *g* for 30 min. * Cryopreserved marrow cells. Negative values are specific cytotoxicities below baseline.

TABLE V.—*K-cell-mediated cytotoxic activity (as in Table IV) of selected AML⁺ antisera against leukaemic myeloblasts, remission lymphocytes and remission bone marrow from a single donor*

| Target → | Myeloblast | *PHA-stimulated blood lymphocytes | Remission blood lymphocytes | Remission marrow |
|---|---|---|---|---|
| % Cr Release with + ve antibody | 70 | 53 | 64 | 53 |
| Baseline release | 20 | 33 | 20 | 11 |
| Serum | | | | |
| 1 | 31+ | 27+ | 2 | 13+ |
| 2 | 18+ | 15+ | −6 | 6 |
| 3 | 15+ | 5 | −4 | 1 |
| 4 | 15+ | 21+ | 0 | 9+ |
| 5 | 14+ | 2 | −11 | 3 |
| 6 | 14+ | −11 | −7 | 3 |
| 7 | 14+ | 35+ | −6 | 7+ |
| 8 | 13+ | 31+ | 7 | 6+ |
| 9 | 12+ | 8+ | 11+ | 1 |
| 10 | 11+ | 29+ | −1 | 5 |
| 11 | 10+ | 20+ | 5 | 8+ |
| 12 | 9+ | 8+ | 15+ | 5+ |
| 13 | 6+ | 7+ | −9 | 6+ |
| 14 | 6+ | −2 | 17+ | 0 |
| 15 | 5+ | −6 | −9 | 5+ |
| 16 | 4+ | 14+ | −11 | 1 |
| 17 | 2+ | 0 | −11 | 2 |

FIG. 3a.

FIG. 3a, b and c.—Binding of absorbed AML⁺ antisera to leukaemic myeloblasts and PHA-stimulated remission cells from 3 donors, before and after absorption with normal bone marrow. The antisera were tested against equal numbers of target myeloblasts and target remission cells in the same experiment. ● ○ activity against the myeloblasts; ■ □ activity against the PHA-stimulated remission cells; ●——■ activity before the marrow absorption; ○ - - □ activity after the marrow absorption. The control antisera which had all been absorbed × 5 with cadaveric spleen were: A: anti-lymphocytic antiserum. B: anti-marrow serum. C: anti-C3H mouse strain-L fibroblast. Lines which are longer than the control antisera indicate increased specificity for the leukaemic myeloblasts. In Fig. 3a these are shown by sera 1, 4, 6, 8, 11, 13 and 15 before absorption with marrow, and serum 2 after absorption. In Fig. 3b, these are sera 1, 2, 4, 5, 6, 12, 14 and 16 before absorption and serum 6 also after absorption, but this is not seen against the myeloblasts of 3a and 3c. In Fig. 3c only sera 13 and 14 show increased activity against AML blasts before absorption.

20

18

16

14

12

10

8

6

4

2

0

ct/min × 10⁻³

A B C

1 2 3 4 5 6 7 8 9 10 11 12 13 14 15 16 17

Controls

Serum number

Fig. 3b.

24

22

20

18

16

14

12

10

8

6

4

2

0

ct/min × 10⁻³

A B C

1 2 3 4 5 6 7 8 9 10 11 12 13 14 15 16 17

Controls

Serum no.

Fig 3c.

different approach was therefore adopted. This was to test the antisera against leukaemic myeloblasts, remission lymphocytes, PHA-stimulated lymphoblasts and remission marrow cells, all from the same donor. This was to avoid differences in polymorphic antigens between leukaemic and normal cells, although it might be argued that the myeloblasts might possess D locus antigens only expressed on a minor fraction of the blood lymphocytes and almost none on the lymphoblasts. Results of a series of this type are shown on Table V. A number of points emerge from this experiment: (1) that sera 3, 5 and 6 induce significant K-cell-mediated cytolysis against myeloblasts and not against the non-leukaemic cells; (ii) that several sera show activity against PHA-stimulated lymphoblasts, but not against lymphocytes; (iii) that marrow cells are relatively unaffected by these antisera.

*The effect of marrow absorption*

The last experiment described above gives some indication that antibody might exist in some of the sera which have specificity, as assessed by K-cell-mediated cytotoxicity, against leukaemic myeloblasts. Normal myeloblasts, however, are a minority population in marrow and cytotoxic assays on the whole marrow might not detect activity against them. Consequently, we subjected all 17 of the sera to 2 absorptions against equal volumes of normal marrow. This resulted in complete loss of selective activity against myeloblasts and PHA-stimulated remission lymphocytes from the same patient. We concluded at this stage that the K-cell-mediated cytotoxicity assay was detecting specificity in these antisera which was directed only against normal tissue antigens.

*The $^{125}I$-anti-immunoglobulin assay used to assess the activity in the selected $AML^+$ antisera*

The same principle as used above (the testing of myeloblasts in parallel with PHA-stimulated lymphoblasts from the same donor) was adopted for assessing antisera with a radioactive anti-rabbit immunoglobulin reagent ($^{125}$I-HAR). Three pairs of myeloblasts and autologous lymphoblasts were tested for antigens which bound the 17 antisera both before and after double marrow absorption. The results are shown in Fig. 3a, b and c. We have drawn the following conclusions from these experiments: (i) Many of the sera before marrow absorption show evidence of activity against myeloblasts, and in some cases also against PHA-induced lymphoblasts from one or 2 donors; (ii) only Serum 13, and then only before marrow absorption, shows clear evidence for activity against an antigen present on myeloblasts from all 3 donors; (iii) a number of the sera show a greater difference between myeloblast and lymphoblast counts before than after absorption against normal marrow. This finding is consistent with the presence of differentiation antigens characteristic of leukaemic myeloblasts and of a minority population in marrow; (iv) there is evidence for polymorphism in these myeloid differentiation antigens, in that the sera show a varying degree of differential activity between the 3 myloblast targets; (v) there is little or no evidence of leukaemia-specific activity.

### DISCUSSION

Contrary to the reports of several other workers (Mohanakumar *et al.*, 1974; Mann *et al.*, 1974; Baker and Taub, 1973; Durantez *et al.*, 1976), our results do not provide support for the concept of immunogenic leukaemia-specific antigens in AML. Despite the exhaustive testing of a large number of heterologous antisera, produced in a variety of ways, we have not been able to develop an antiserum which has potent activity against leukaemic myeloblasts but no significant activity against normal tissues. Although the AML titre of the antisera before absorption varied from 1/39,000 to 1/300,000, all the activity could be removed by absorption with splenic lymphocytes and marrow cells. Splenic lymphocytes alone were able to remove much of this activity, indicating that common antigens between lymphocytes and leukaemic myeloblasts are responsible for most of the anti-AML activity in the AML antisera. Fu *et al.* (1975), Billing *et al.* (1976; 1977) reported that a high proportion of undifferentiated lymphoblastic and myeloblastic leukaemias react with antisera directed against human Ia-like antigens. These HL-A D-locus antigens are not present on normal blood T cells or T blasts. Consequently, assays with a high cut-off point for cytotoxicity will find anti-Ia sera negative for blood lymphocytes but positive for most myeloid leukaemic cells. This might explain much of the activity which was present in previously reported anti-leukaemic antisera. Similarly, antisera raised with cells from B lymphoblastoid cell lines (*e.g.* RAJI) (Mann *et al.*, 1971; 1974; 1975*b*; Durantez *et al.*, 1976) as well as spontaneous anti-leukaemic antibody found in normal individuals (Bias *et al.*, 1972; Cullen and Mason, 1976) might be attributable to these antigens. Despite the large number of reports suggesting that leukaemia-specific antigens exist, some investigators have failed to find antibody specific for antigens found exclusively on AML blasts, for example, in human subjects undergoing active immunotherapy (Gale and MacLennan, 1977; Klouda *et al.*, 1975). Also, one worker raising antisera against AML blasts (Greaves, 1975) failed to confirm the presence of tumour-specific antigens in this disease.

Analysis of the AML antisera in this study, therefore, reveals that they recognize cell-surface antigens on leukaemic myeloblasts which are essentially, if not completely, equivalent to normal antigens found on splenic lymphocytes and marrow cells. This includes activity against human alloantigens as well as against cell-cycle-associated antigens present on PHA-stimulated remission lymphocytes but not on unstimulated cells. The increase in reactivity against PHA-stimulated cells was not explained by increased susceptibility to cytotoxic lysis, since this activity was also shown by the $^{125}$iodinated anti-immunoglobulin assay. Only a small fraction of the total activity of the antisera was directed against putative myeloid differentiation antigen, suggesting that they may not be prominent on the cell-surface of these cells.

Marrow was extremely effective in removing residual activity (against both leukaemic myeloblasts and PHA-induced lymphoblasts) which remained after absorption with splenic lymphocytes. Spleen cells were used because of the availability of this tissue at post-mortem, and the presence of a high proportion of B cells expressing HL-A D-locus antigens and mature myeloid cells. Because of the heterogeneity of cells in marrow preparations, it is difficult to specify from these studies which cells were particularly responsible for removing the residual anti-myeloblastic activity. However, failure to absorb activity with spleen militates against this activity being on a mature myeloid cell. The low level of cytotoxicity induced against marrow cells by the 17 final antisera, before their absorption with marrow, suggests that the absorbing antigen in marrow is on a minority of cells. The antisera which showed most residual activity against AML blasts after spleen tissue absorption were produced: (1) following modification of the immunizing cells with neuraminidase-treatment of NABS-coating, or (2) by immunization of animals in which induction of tolerance against normal antigens had been attempted. Detection of these antigens may be useful for diagnostic purposes, and could help in distinguishing variants of the acute myeloblastic leukaemias. At the moment we are attempting to identify the nature of the normal marrow cells which show antigens common with leukaemic myeloblast but not found in splenic tissue. We hope that the antisera absorbed with spleen will help to provide markers for the physiological stages of myeloid differentiation.

One of the most striking positive findings in this study has been the high level of reactivity of antisera against allo-antigens. To some extent this may reflect the use of the K-cell cytotoxicity assay. This assay measures IgG antibody (MacLennan, 1972) whereas complement-dependent cytolysis is activated more efficiently on a molar basis by IgM than IgG (Humphrey, 1967; Linscott, 1970). The early screening of absorbed antisera in this study included complement-dependent as well as K-cell-dependent cytolysis. However, use of the former was discontinued after the second spleen cell absorption, as nearly all complement-revealed activity had been lost at that stage (Tupchong, 1978). Rabbits have been used previously to raise antibody against human polymorphic antigens (Einstein *et al.*, 1971), but, it does seem that further use of the rat K-cell assay for detecting xeno-antibodies against human HL-A antigens may be profitable.

### REFERENCES

Arbeit, R. D., Sachs, D. H., Amos, D. B. & Dickler, H. B. (1975) Human lymphocyte allo-antigen(s) similar to murine Ir-region-associated (1a) antigens. *J. Immunol.*, **115**, 1173.

Avrameas, S. & Ternynck, T. (1969) The cross-linking of proteins with glutaraldehyde and its use for the preparation of immunoadsorbents. *Immunochemistry*, **6**, 53.

Baker, M. A. & Taub, R. N. (1973) Production of antiserum in mice to human leukaemia-associated antigens. *Nature* (*New Biology*), **241**, 93.

Baker, M. A., Taub, R. N., Brown, S. M. & Ramachandar, K. (1974) Delayed cutaneous hypersensitivity in leukaemic patients to autologous blast cells. *Br. J. Haematol.*, **27**, 627.

Bias, W. B., Santos, G. W. & Burke, P. J. (1972) Cytotoxic antibody in normal human serums reactive with tumour cells from acute lymphocytic leukaemia. *Science*, **178**, 304.

Billing, R. J., Rafizadeh, B., Drew, I., Hartman, G., Gale, B. & Tersaki, P. I. (1976) Human B-lymphocyte antigens expressed by lymphocytic and myelocytic leukaemia cells: I. Detection by rabbit antisera. *J. exp Med.*, **144**, 167.

Billing, R. J., Ting, A. & Terasaki, P. I. (1977) Human B-lymphocyte antigens expressed by lymphocytic and myelocytic leukaemia cells: II. Detection by human anti-B-cell alloantisera. *J. Natl. Cancer Inst.*, **58**, 199.

Boyum, A. (1968) Separation of leukocytes from blood and bone marrow. *Scand. J. Clin. Lab. Invest.*, **21**, (Suppl. 97), 31.

Cresswell, P. & Geier, S. S. (1975) Antisera to human B lymphocyte membrane glycoproteins block stimulations in mixed lymphocyte culture. *Nature*, **257**, 147.

Cullen, P. R. & Mason, D. Y. (1976) Leukaemia-associated antigens in man. Detection by antibodies in maternal sera. *Clin exp. Immunol.*, **17**, 571.

Durantez, A., Zighelboim, J. & Gale, R. P. (1976) Leukaemia-associated antigens detected by heterologous antisera. *J. Natl. Cancer Inst.*, **56**, 1217.

Einstein, A. B., Mann, D. L., Gordon, H. C., Trapani, R. J. & Fahey, J. L. (1971) Heterologous antisera against specific HL-A antigens. *Transplantation*, **12**, 299.

Fefer, A., Michelson, E. & Thomas, E. D. (1976) Stimulation of lymphocytes in mixed culture by cells from HL-A identical siblings. *Clin. exp. Immunol.*, **23**, 214.

Freeman, C. B., Harris, R., Geary, C. G., Leyland, M. J., McIver, J. E. & Delamore, I. W. (1973) Active immunotherapy used alone for maintenance of patients with acute myeloid leukaemia. *Br. med. J.*, **4**, 571.

Fridman, W. H. & Kourilsky, F. M. (1969) Stimulations of lymphocytes by autologous leukaemic cells in acute leukaemia. *Nature*, **224**, 277.

Fu, S. M., Winchester, R. J. & Kunkel, H. G. (1975) The occurrence of HL-B alloantigens on the cells of unclassified acute lymphoblastic leukaemias *J. exp. Med.*, **142**, 1334.

Gale, D. G. & MacLennan, I. C. M. (1977) Cytotoxic antibody in acute myeloblastic leukaemia during immunotherapy: Lack of tumour specificity. *Br. J. Cancer*, 35, 280.

Garb, S., Stein, A. A. & Sims, G. (1962) The production of antihuman leukaemia serum in rabbits. *J. immunol*, **88**, 142.

Greaves, M. F. (1975) Clinical applications of cell-surface markers. *Prog. Haematol.*, **9**, 255.

Hayhoe, F. G. J., Quaglino, D. & Doll, R. (1964) The cytology and cytochemistry of acute leukaemias. A study of 140 cases. *M.R.C. Special Rep. Series*, No. 304. H.M.S.O.

Humphrey, J. H. (1967) Haemolytic efficiency of rabbit IgG anti-Frossman antibody and its augmentation by anti-rabbit IgG. *Nature*, **216**, 1295.

Hyde, R. M., Garb, S. & Bennett, A. J. (1967) Demonstration by immunoelectrophoresis of antigen in human myelogenous leukaemia. *J. Natl. Cancer Inst.*, **38**, 909.

Jensenius, J. C. & Williams, A. F. (1974) The binding of anti-immunoglobulin antibodies to rat thymocytes and thoracic duct lymphocytes. *Eur. J. Immunol.*, **4**, 91.

Klouda, P. T., Lawler, S. D., Powles, R. L., Oliver, R. T. D. & Grant, C. K. (1975) HL-A antibody response in patients with acute myelogenous leukaemia treated by immunotherapy *Transplantation*, **19**, 245.

Kuntz, M. M., Innes, J. B. & Weksler, M. E. (1976) Lymphocyte transformation induced by autologous cells: IV. Lymphocyte proliferation induced by autologous or allogeneic non-T lymphocytes. *J. exp. Med.*, **143**, 1042.

Linscott, W. D. (1970) The effect of cell surface antigen density on immunological enhancement. *Nature*, **288**, 824.

Lohrmann, H. P., Novikov, L. & Graw, R. G. (1974) Stimulatory capacity of human T and B

lymphocytes in mixed leukocyte cultures. *Nature*, **250**, 144.

MacLennan, I. C. M. (1972) Antibody in the induction and inhibition of lymphocyte cytotoxicity *Transplant. Revs.*, **13**, 67.

Mann, D. L., Rogentine, G. N., Halterman, R. & Leventhal, B. (1971) Detection of an antigen associated with acute leukaemia. *Science*, **174**, 1136.

Mann, D. L., Halterman, R. & Leventhal, B. (1974) Acute leukaemia-associated antigens. *Cancer* **34**, (Suppl.), 1446.

Mann, D. L., Abelson, L., Harris, S. & Amos, D. B. (1975*a*) Detection of antigens specific for B-lymphoid culture cell lines with human alloantisera. *J. exp. Med.*, **142**, 84.

Mann, D. L., Leventhal, B. & Halterman, R. (1975*b*) Human antisera detecting leukaemia-associated antigens on autochthonous tumour cells. *J. Natl. Cancer Inst.*, **54**, 345.

Metzgar, R. S., Mohanakumar, T. & Miller, D. S. (1972) Antigens specific for human lymphocytic and myeloid luekaemia cells: detection by non-human primates. *Science*, **178**, 986.

Mohanakumar, T., Metzgar, R. S. & Miller, D. S. (1974) Human leukaemia cell antigens: Serologic characterisation with xenoantisera. *J. Natl. Cancer Inst.*, **52**, 1435.

Opelz, G., Kiuchi, M., Takasugi, M. & Terasaki, P. I. (1975) Autologous stimulation of human lymphocyte subpopulations. *J. exp. Med.*, **142**, 1327.

Opelz, G., Gale, P. R. & McClelland, J. D. (1977) Relationship between leukaemia antigens and stimulation in mixed leukocytes cultures. *J. Natl. Cancer Inst.*, **59**, 95.

Powles, R. L., Balchin, L. A., Hamilton-Fairley, G. & Alexander, P. (1971) Recognition of leukaemic cells as foreign before and after autoimmunization. *Br. Med. J.*, **1**, 486.

Powles, R. L., Crowther, D., Bateman, C. J. T. & 12 others (1973) Immunotherapy for acute myelogenous leukaemia. *Br. J. Cancer*, **28**, 365.

Powles, R. L., Russell, J., Lister, T. A., and 6 others (1977) Immunotherapy for acute myelogenous leukaemia: a controlled clinical study 2½ years after entry of last patient. *Br. J. Cancer*, **35**, 265.

Reisfeld, R. A. & Kahn, B. D. (1971) Extraction and purification of soluble histocompatibility antigens. *Transplant. Rev.*, **6**, 81.

Schlossmann, S. F., Chess, L., Humphreys, R. E. & Strominger, J. L. (1976) Distribution of Ia-like molecules on the surface of normal and leukaemic human cells. *Proc. Natl. Acad. Sci.*, **73**, 1288.

Taylor, G. M., Freeman, C. B. & Harris, R. (1976) Response of remission lymphocytes to autochthonous leukaemic myeloblasts. *Br. J. Cancer*, **33**, 501.

Tupchong, L. (1978) *Surface antigens in acute myeloblastic leukaemia: a study using heterologous antisera.* DPhil Thesis, University of Oxford.

Viza, D., Bernard-Degani, O., Bernard, C. & Harris, R. (1969) Leukaemic antigens. *Lancet*, ii, 393.

Waller, C. A. & MacLennan, I. C. M. (1977) Analysis of lymphocytes in blood and tissue. In *Clinical Immunology*, Ed. R. Thompson, Oxford: Blackwells. p. 170.

Zighelboim, J., Bick, A. & Durantez, A. (1977) Recognition by human and rabbit sera of common antigens to leukaemia blast cells, peripheral blood B-lymphocytes and monocytes. *Cancer Res.*, **37**, 3656.